# MFST-GCN: A Sleep Stage Classification Method Based on Multi-Feature Spatio-Temporal Graph Convolutional Network

**DOI:** 10.3390/brainsci16020162

**Published:** 2026-01-30

**Authors:** Huifu Li, Xun Zhang, Ke Guo

**Affiliations:** 1 School of Computer and Artificial Intelligence, Beijing Technology and Business University, Beijing 100048, China; 2 School of Mathematics and Physics, Xinjiang Hetian College, Hetian 848000, China; 3 Beijing Laboratory for System Engineering of Carbon Neutrality, Beijing 100081, China

**Keywords:** sleep stage classification, sleep functional connectivity, graph connection network, multi-scale attention network

## Abstract

**Background/Objectives:** Accurate sleep stage classification is essential for evaluating sleep quality and diagnosing sleep disorders. Despite recent advances in deep learning, existing models inadequately represent complex brain dynamics, particularly the time-lag effects inherent in neural signal propagation and regional variations in cortical activation patterns. **Methods:** We propose the MFST-GCN, a graph-based deep learning framework that models these neurobiological phenomena through three complementary modules. The Dynamic Dual-Scale Functional Connectivity Modeling (DDFCM) module constructs time-varying adjacency matrices using Pearson correlation across 1 s and 5 s windows, capturing both transient signal transmission and sustained connectivity states. This dual-scale approach reflects the biological reality that neural information propagates with measurable delays across brain regions. The Multi-Scale Morphological Feature Extraction Network (MMFEN) employs parallel convolutional branches with varying kernel sizes to extract frequency-specific features corresponding to different EEG rhythms, addressing regional heterogeneity in neural activation. The Adaptive Spatio-Temporal Graph Convolutional Network (ASTGCN) integrates spatial and temporal features through Chebyshev graph convolutions with attention mechanisms, encoding evolving functional dependencies across sleep stages. **Results:** Evaluation on ISRUC-S1 and ISRUC-S3 datasets demonstrates F1-scores of 0.823 and 0.835, respectively, outperforming state-of-the-art methods. **Conclusions:** Ablation studies confirm that explicit time-lag modeling contributes substantially to performance gains, particularly in discriminating transitional sleep stages.

## 1. Introduction

Sleep is a fundamental physiological process critical for cognitive function, emotional regulation, and physical health [1]. Epidemiological studies link poor sleep quality to cardiovascular diseases, metabolic disorders, and neurological conditions [2,3]. Polysomnography (PSG) remains the clinical standard for sleep assessment [4,5], requiring manual classification of recordings into stages (Wake, N1, N2, N3, REM) according to American Academy of Sleep Medicine criteria [6]. However, manual annotation is labor-intensive and subject to inter-rater variability, particularly during ambiguous transition periods. Automated sleep staging algorithms offer an objective, scalable alternative with significant clinical potential.

Deep learning approaches have progressively improved automated sleep staging. Early methods employed Convolutional Neural Networks (CNNs) to extract spatial features from EEG signals or recurrent neural networks to model temporal dependencies [7,8]. While effective, these architectures treat multichannel EEG as independent streams or flattened grids, neglecting the topological organization of brain networks. Graph Convolutional Networks (GCNs) have emerged to address this limitation by explicitly modeling spatial relationships between EEG channels. Recent work, such as STRFLNet [9] and the CR-GCN [10], demonstrates that incorporating graph-structured representations improves classification by capturing inter-regional dependencies.

Despite these advances, a critical neurobiological phenomenon remains underexplored: neural signals propagate with finite velocity, introducing temporal delays in information transmission between brain regions. Most GCN-based models assume instantaneous connectivity, constructing static or time-invariant adjacency matrices that fail to account for propagation latencies [11]. This simplification is problematic during rapid stage transitions (e.g., N1 to N2), where temporal misalignments in functional connectivity critically influence classification accuracy. Additionally, existing models often overlook regional heterogeneity—different cortical areas exhibit distinct activation patterns and frequency characteristics across sleep stages, necessitating adaptive feature extraction mechanisms.

To address these gaps, we propose the MFST-GCN (Multi-Scale Functional Spatio-Temporal Graph Convolutional Network), a framework designed around three biologically motivated components:

- Dynamic Dual-Scale Functional Connectivity Modeling (DDFCM): This module constructs time-varying adjacency matrices by computing Pearson correlations across short-term (1 s) and long-term (5 s) windows. Short-term connectivity captures rapid, transient signal transmission, while long-term connectivity reflects sustained neural states. This dual-scale approach explicitly models propagation delays, improving sensitivity to temporal dynamics during stage transitions.
- Multi-Scale Morphological Feature Extraction Network (MMFEN): Parallel convolutional branches with kernel sizes spanning 1 to 7 extract features corresponding to different EEG frequency bands (delta, theta, alpha, beta). This multi-resolution design addresses regional heterogeneity by adaptively weighting frequency-specific components relevant to each sleep stage.
- Adaptive Spatio-Temporal Graph Convolutional Network (ASTGCN): Chebyshev graph convolutions combined with temporal and spatial attention mechanisms integrate features from DDFCM and the MMFEN. This module encodes how functional connectivity evolves across sleep cycles, enabling robust classification under inter-subject variability.

## 2. Related Works

Automated sleep stage classification has evolved from manual feature engineering to end-to-end deep learning systems, driven by clinical needs to reduce inter-rater variability and enable large-scale sleep studies. We organize existing methods into three categories based on their modeling assumptions: temporal sequence modeling, multi-scale feature extraction, and graph-based spatial modeling.

### 2.1. Temporal Sequence Models

Sleep exhibits structured temporal dynamics across multiple scales: rapid transitions between micro-sleep events (K-complexes, sleep spindles) and gradual progression through 90 min ultradian cycles. Early deep learning approaches applied Recurrent Neural Networks (RNNs) to model these sequential dependencies [12]. SingleChannelNet [13] combined multi-scale convolutions with temporal pooling to capture epoch-to-epoch transitions. LSTM-based architectures further refined this by explicitly modeling long-range dependencies across consecutive 30 s epochs [7], while GRU variants focused on macro-structural sleep patterns [8].

These models treat sleep as a Markov chain where each epoch depends only on previous epochs, assuming instantaneous neural state transitions. However, neurophysiological studies demonstrate that sleep-related brain activity propagates across cortical regions with measurable delays. Slow-wave oscillations during N3 sleep, for example, originate in frontal regions and propagate posteriorly with phase lags detectable via EEG. Current sequential models process each 30 s window as an atomic unit, failing to represent these within-epoch propagation dynamics. This limitation is particularly problematic during transitional stages where transient connectivity patterns are diagnostically informative. Moreover, propagation delays are altered in neurological conditions such as traumatic brain injury and Alzheimer’s disease, suggesting that models capturing these dynamics could enable earlier detection of pathological sleep patterns.

### 2.2. Multi-Scale Feature Extraction

EEG signals are inherently multi-scale, containing rhythms from delta (0.5–4 Hz) to gamma (>30 Hz) bands that carry distinct physiological meanings. Multi-scale convolutional architectures decompose these frequency components to improve classification. The MS-HNN [14] employed parallel convolutional branches with Squeeze-and-Excitation modules to extract band-specific features from single-channel recordings. Multi-channel methods like MultiChannelSleepNet [15] and the MSLFE [16] extended this approach by applying multi-kernel convolutions across spatial dimensions to capture local topographic patterns [17].

While these models extract multi-scale features effectively, they apply uniform processing across all channels, implicitly assuming spatial homogeneity. This contradicts established sleep neurophysiology: N2 sleep spindles are most prominent in central electrodes (C3/C4), while N3 slow waves dominate frontal regions (F3/F4). REM sleep exhibits occipital alpha dominance, whereas N1 shows diffuse theta activity. Existing multi-scale models lack channel-specific attention mechanisms to adaptively weight these regionally heterogeneous signals. Regional sleep abnormalities serve as biomarkers for neuropsychiatric disorders—reduced frontal slow-wave activity characterizes depression while focal sleep disturbances indicate epileptic foci. Models respecting regional heterogeneity could therefore improve diagnostic specificity.

### 2.3. Graph Convolutional Networks

GCNs have emerged to model spatial dependencies between EEG channels as nodes in a graph. Early work defined adjacency matrices based on Euclidean distances between electrode positions [18]. Recent advances construct data-driven graphs: the CR-GCN [10] learned channel relationships for emotion recognition, while STRFLNet [9] fused spatial and temporal graph representations for global dependency modeling.

Current GCN methods construct adjacency matrices using zero-lag correlations, assuming that functional connections are instantaneous. This conflicts with functional connectivity studies showing that brain regions interact with time-delayed coupling. Thalamocortical loops during N2 sleep, for instance, exhibit oscillatory phase lags between the thalamus and cortex that generate sleep spindles. Static or single-timescale graphs cannot represent these lagged interactions, which are critical for distinguishing between stages with similar spectral content but different connectivity patterns (e.g., N2 vs. N3). Additionally, most GCN sleep staging works use fixed graphs or update adjacency at coarse temporal scales (per-subject or per-night), failing to capture rapid connectivity reconfigurations during stage transitions [11].

No existing sleep staging framework simultaneously addresses within-epoch propagation delays via multi-timescale connectivity, regional activation heterogeneity through adaptive channel attention, and rapid connectivity dynamics through fine-grained temporal graphs. Our MFST-GCN framework explicitly targets these gaps through a neurobiologically motivated design: DDFCM constructs 1 s and 5 s lagged correlation graphs to capture both rapid and sustained connectivity; the MMFEN with channel-specific attention enables adaptive weighting of regional frequency signatures; and the ASTGCN integrates these components through time-varying graph convolutions. This positions our work as bridging the gap between instantaneous computational models and time-lagged neurophysiological reality.

## 3. Materials and Methods

### 3.1. Architecture Overview

**Figure 1:**
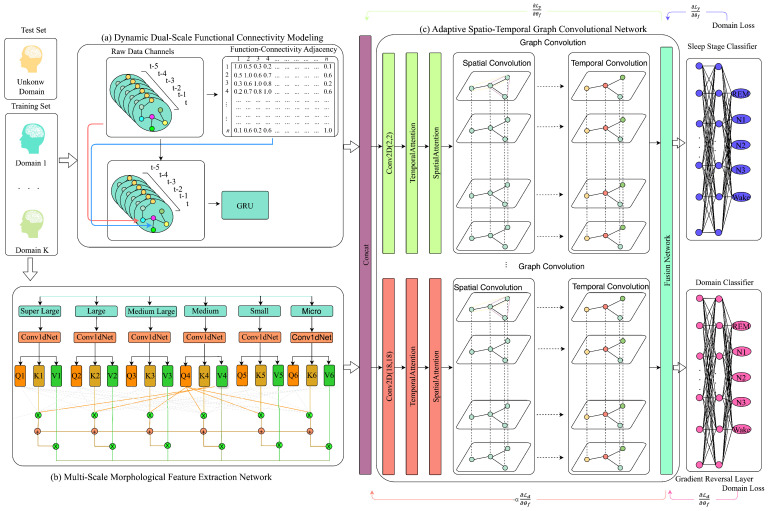
Overall architecture of MFST-GCN. Raw EEG (6 channels × 6000 samples per 30 s epoch) is processed through two parallel pathways: (1) DDFCM constructs dual-scale adjacency matrices via Pearson correlation over 1 s ($A_{fast}$) and 5 s ($A_{slow}$) windows, with temporal evolution encoded by GRU to produce connectivity embedding $H_{conn}$ (128-dim); (2) MMFEN applies six parallel 1D convolutional branches with kernel sizes k∈{50,100,200,400,700,1000} samples, refined by multi-scale attention to produce morphological features $H_{morph}$ (384-dim). ASTGCN integrates concatenated features $\left[H_{morph}⊕H_{conn}\right]$ (512-dim) using Chebyshev graph convolutions with spatial and temporal attention, followed by Mixture of Experts fusion to generate final embedding (256-dim). ADG with gradient reversal layer enforces subject invariance during training. Output layer produces sleep stage probabilities for Wake, N1, N2, N3, and REM.

The MFST-GCN framework addresses three neurobiological phenomena critical for sleep stage classification: neural signal propagation delays, regional heterogeneity in cortical activation, and dynamic evolution of functional connectivity. As illustrated in Figure 1, the architecture comprises three synergistic modules with distinct computational roles (Table 1).

Dynamic Dual-Scale Functional Connectivity Modeling (DDFCM) constructs time-varying brain graphs using correlation windows of 1 s and 5 s. These timescales were selected to match the characteristic durations of sleep-relevant neural processes: rapid spindle–thalamocortical coupling (0.5–2 s) and sustained slow-wave propagation (2–4 s) [19,20]. This dual-scale design captures both transient signal transmission and homeostatic connectivity states, explicitly modeling the propagation delays that zero-lag correlation methods ignore.

The MMFEN employs six parallel convolutional branches with kernel sizes ranging from 50 to 1000 samples (0.25 to 5 s at 200 Hz sampling). This multi-resolution architecture corresponds to different EEG frequency bands: smaller kernels detect rapid transients in beta/gamma ranges, while larger kernels capture sustained delta/theta oscillations. Parallel processing with adaptive attention addresses regional heterogeneity by allowing the model to weight channel-specific frequency components.

The ASTGCN integrates connectivity patterns from DDFCM and morphological features from the MMFEN through Chebyshev graph convolutions combined with spatial and temporal attention mechanisms. A Mixture of Experts fusion strategy dynamically weights the six feature streams based on their relevance to each sleep stage.

Finally, Adversarial Domain Generalization (ADG) enforces subject-invariant representations through gradient reversal, addressing inter-individual variability in EEG baseline characteristics to improve generalization to unseen subjects.

### 3.2. Dynamic Dual-Scale Functional Connectivity Modeling

Neurophysiological evidence demonstrates that brain regions communicate with finite propagation velocities, introducing temporal delays in functional coupling. Thalamocortical loops during N2 sleep, for instance, exhibit phase lags of 10–100 ms between the thalamus and cortex during sleep spindle generation. Traditional graph neural networks construct adjacency matrices using zero-lag correlations, assuming instantaneous connectivity that fails to represent these propagation dynamics.

Traditional graph neural networks often rely on static adjacency matrices, which fail to capture the “time-lag effect” inherent in neural information transmission. To mathematically model this propagation delay, the DDFCM module constructs a dynamic graph G=(V,E), where V represents the set of *n* EEG channels. We propose a dual-scale strategy that generates two distinct connectivity matrices, $A_{fast}$ and $A_{slow}$, based on a 1 s immediate window (t−1) and a 5 s sustained window (t−5 to t−1), respectively. This multi-temporal design effectively captures both rapid transient responses and homeostatic functional stability. We quantify the functional synchronization between any two channels *i* and *j* using the Pearson correlation coefficient. For a given time window *T*, the connection weight $p_{i,j}$ is formalized as

(1) $$p_{i,j}=\frac{\sum_{k=1}^{T}\left(x_{i,k}-\mu_{i}\right)\left(x_{j,k}-\mu_{j}\right)}{\sqrt{\sum_{k=1}^{T}{\left(x_{i,k}-\mu_{i}\right)}^{2}}\sqrt{\sum_{k=1}^{T}{\left(x_{j,k}-\mu_{j}\right)}^{2}}}$$

where $x_{i,k}$ denotes the signal amplitude of channel *i* at time step *k*. To synthesize a comprehensive topology, we define a fused edge weight $e_{i}^{\prime }$ for node *i* by integrating contributions from both temporal scales via learnable importance parameters α and β. The fusion process effectively acts as an adaptive message-passing mechanism:

(2) $$e_{i}^{\prime }=x_{i}^{\left(t\right)}+\sum_{j\in N(i)}\left(\alpha_{j}\cdot I\left(p_{ij}\in A_{slow}\right)\cdot x_{j}^{\left(t\right)}+\beta_{j}\cdot I\left(p_{ij}\in A_{fast}\right)\cdot x_{j}^{\left(t\right)}\right)$$

In this formulation, I(·) serves as an indicator function to enforce topological sparsity. The temporal evolution of this dynamic graph is subsequently encoded by a Gated Recurrent Unit (GRU). To rigorously capture the sequential dependencies, the update gate $z_{t}$ and reset gate $r_{t}$ at time *t* are calculated as

(3) $$z_{t}=\sigma \left(W_{z}e_{t}^{\prime }+U_{z}h_{t-1}+b_{z}\right)$$

(4) $$r_{t}=\sigma \left(W_{r}e_{t}^{\prime }+U_{r}h_{t-1}+b_{r}\right)$$

The hidden state $h_{t}$ is then updated by balancing the historical information and the current candidate state:

(5) $$h_{t}=\left(1-z_{t}\right)⊙h_{t-1}+z_{t}⊙\tanh\left(W_{h}e_{t}^{\prime }+U_{h}\left(r_{t}⊙h_{t-1}\right)+b_{h}\right)$$

This architecture ensures that the model adaptively retains critical historical connectivity patterns while discarding transient artifacts, producing the final connectivity embedding $H_{conn}$.

### 3.3. Multi-Scale Morphological Feature Extraction Network

**Figure 2:**
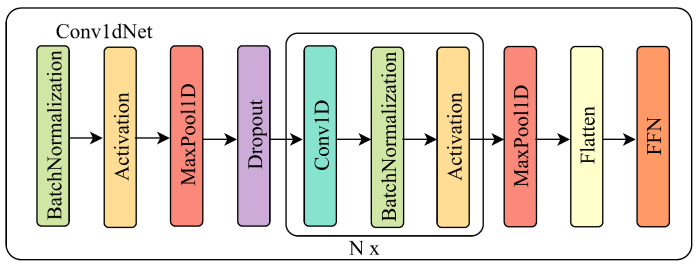
Architecture of individual Conv1dNet branch in MMFEN. Each branch processes input EEG through sequential operations: (1) 1D convolution with branch-specific kernel size (ranging from 50 to 1000 samples), (2) batch normalization for stable training, (3) ReLU activation for nonlinearity, (4) max pooling with stride 2 for dimensionality reduction. Smaller-kernel branches (50–100 samples) are optimized to detect rapid transients such as K-complexes and spindle bursts, corresponding to beta and sigma frequency bands. Larger-kernel branches (700–1000 samples) capture sustained oscillatory patterns characteristic of delta and theta slow-wave activity. Each branch outputs a feature map encoding patterns at its specific temporal scale, which are subsequently refined by the multi-scale attention mechanism before concatenation.

Sleep biomarkers manifest across diverse temporal scales reflecting distinct neurophysiological processes. K-complexes span 0.5–2 s and sleep spindles persist for 0.5–1.5 s, while slow waves extend 2–4 s [19]. A single convolutional kernel cannot simultaneously resolve these phenomena due to fixed receptive field limitations. On this basis, the MMFEN is proposed, and the specific structure of this module is shown in Figure 2.

The MMFEN employs six parallel convolutional branches with increasing temporal receptive fields designed to match characteristic durations of EEG rhythms. Branch kernel size k∈{50,100,200,400,700,1000} samples correspond to temporal windows of 0.25, 0.5, 1, 2, 3.5, and 5 s at a 200 Hz sampling rate, spanning from high-frequency beta oscillations to low-frequency delta waves. For the *k*-th branch with kernel $\Psi_{k}$, the convolution operation on input X is

(6) $$M_{k}\left(t\right)=\left(X*\Psi_{k}\right)\left(t\right)=\sum_{\tau }X\left(t-\tau \right)\cdot \Psi_{k}\left(\tau \right)$$

To dynamically synthesize these multi-view representations, we employ a multi-scale self-attention mechanism. We compute the Query (Q), Key (K), and Value (V) projections by mapping the feature map $M_{k}$ into a shared latent subspace via learnable matrices $W_{Q},W_{K},W_{V}$:

(7) $$Q_{k}=M_{k}W_{Q}+b_{Q}$$

(8) $$K_{k}=M_{k}W_{K}+b_{K}$$

(9) $$V_{k}=M_{k}W_{V}+b_{V}$$

The attention-refined feature $Z_{k}$ is then derived via a scaled dot-product attention, which allows the model to selectively enhance informative frequency components while suppressing irrelevant noise bands. The calculation is performed as

(10) $$Z_{k}=\mathrm{Softmax}\left(\frac{Q_{k}K_{k}^{⊤}}{\sqrt{d_{k}}}\right)V_{k}$$

where $d_{k}$ is the scaling dimension. The final morphological representation $H_{morph}$ is obtained by the concatenation of all branches:

(11) $$H_{morph}=\mathrm{Concat}\left(Z_{1},Z_{2},\ldots ,Z_{6}\right)$$

This comprehensive feature set provides a high-dimensional spectral profile of the EEG signal across multiple temporal resolutions.

### 3.4. Adaptive Spatio-Temporal Graph Convolutional Network

**Figure 3:**
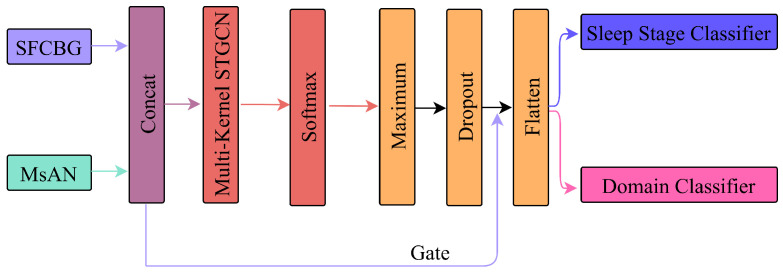
Mixture of Experts fusion architecture in ASTGCN. Six parallel processing branches apply spatio-temporal graph convolutions to features from different morphological scales. Each branch independently processes spatial dependencies (via Chebyshev graph filters) and temporal evolution (via temporal attention), producing expert outputs $Y_{1},\ldots ,Y_{6}$. The gating network (highlighted in red) computes attention weights $G\left(H\right)=\left[g_{1},\ldots ,g_{6}\right]$ by applying softmax to learned projections of concatenated features $H_{joint}$. Final output $Y_{final}=\sum g_{i}Y_{i}$ is a dynamic weighted sum, where weights adapt based on input characteristics.

To unify the topological insights from DDFCM and the morphological features from the MMFEN, the ASTGCN module treats the brain as a dynamic graph evolving in space and time, as illustrated in Figure 3. The joint feature space is initially formed as $H_{joint}=\left[H_{morph}⊕H_{conn}\right]$. We introduce a dual-attention mechanism to capture dynamic correlations. The spatial attention matrix $S_{att}$ determines the significance of inter-channel relationships, while the temporal attention matrix $T_{att}$ captures long-range dependencies between sleep epochs:

(12) $$S_{att}=\mathrm{Softmax}\left(V_{s}\cdot \sigma \left(\left(HW_{1}\right)W_{2}\cdot {\left(W_{3}H\right)}^{T}+b_{s}\right)\right)$$

(13) $$T_{att}=\mathrm{Softmax}\left(V_{t}\cdot \sigma \left(\left(H^{T}U_{1}\right)U_{2}\cdot {\left(U_{3}H^{T}\right)}^{T}+b_{t}\right)\right)$$

The spatial structure is then modeled using spectral graph convolutions approximated by Chebyshev polynomials of order *K*. The recursive definition of the Chebyshev polynomials $T_{k}\left(x\right)$ is given by

(14) $$T_{k}\left(x\right)=2xT_{k-1}\left(x\right)-T_{k-2}\left(x\right)$$

where $T_{0}\left(x\right)=1$ and $T_{1}\left(x\right)=x$. Using this approximation, the graph convolution operation $Y_{spatial}$ for the graph signal H is formalized as

(15) $$Y_{spatial}=\sum_{k=0}^{K-1}\theta_{k}T_{k}\left(\tilde{L}\right)\left(H⊙S_{att}\right)$$

where $\tilde{L}$ is the scaled normalized Laplacian. To robustly fuse the diverse features generated by the parallel multi-kernel streams, we employ a fusion network based on a Mixture of Experts (MoE) strategy. We define a gating network G(H) that outputs a probability distribution over the available experts:

(16) $$G\left(H\right)=\mathrm{Softmax}\left(W_{g}H_{joint}+b_{g}\right)$$

The final integrated output $Y_{final}$ is a weighted combination of all kernels, ensuring the selection of the most discriminative features:

(17) $$Y_{final}=\sum_{i=1}^{6}G{\left(H\right)}_{i}\cdot Y_{i}$$

### 3.5. Adversarial Subject-Invariant Learning

**Figure 4:**
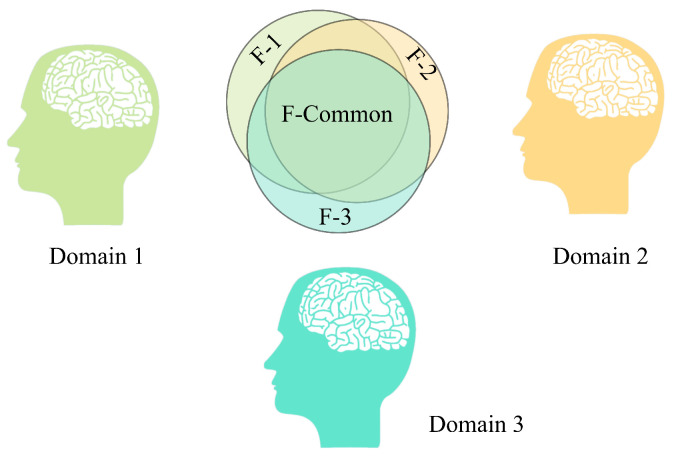
Adversarial domain generalization mechanism for enforcing subject-invariant representations. Feature extractor $G_{f}$ (comprising DDFCM, MMFEN, and ASTGCN modules) processes input EEG to produce embedding F. Two classifiers operate on this embedding: (1) label classifier $G_{y}$ predicts sleep stage (Wake, N1, N2, N3, REM) from F, trained to minimize classification error; (2) domain discriminator $G_{d}$ attempts to identify subject ID from F passed through Gradient Reversal Layer (GRL), which inverts gradients during backpropagation. This adversarial setup creates opposing optimization objectives: $G_{y}$ encourages $G_{f}$ to learn sleep-discriminative features (minimizing $L_{class}$), while $G_{d}$ encourages $G_{f}$ to eliminate subject-specific information (but GRL inverts this to maximize $L_{domain}$). The equilibrium produces F-Common features that are highly discriminative for sleep stages yet invariant to subject identity, enabling robust generalization to unseen patients without domain-specific fine-tuning.

A critical impediment to clinical deployment is the “domain shift” caused by inter-subject variability. We address this via an Adversarial Domain Generalization (ADG) framework, which enforces the model to learn a representation space where source domains are indistinguishable. ADG has broad applicability, with good performance in applications such as fault diagnosis and image classification [21]. Figure 4 illustrates the intuitive concept of adversarial domain generalization. The framework comprises a feature extractor $G_{f}$, a domain discriminator $G_{d}$, and a label classifier $G_{y}$. To effectively remove subject-specific information, we introduce a Gradient Reversal Layer (GRL) denoted as $R_{\lambda }\left(\cdot \right)$. Mathematically, the GRL is defined as an identity transformation during the forward pass and a gradient negation during the backward pass:

(18) $$R_{\lambda }\left(x\right)=x$$

(19) $$\frac{dR_{\lambda }\left(x\right)}{dx}=-\lambda I$$

where λ is a hyperparameter that controls the adversarial strength. The optimization objective is formulated as a minimax game between the feature extractor and the domain discriminator:

(20) $$\min_{\theta_{f},\theta_{y}}\max_{\theta_{d}}L\left(\theta_{f},\theta_{y},\theta_{d}\right)=L_{class}\left(G_{y}\left(G_{f}\left(X\right)\right),y\right)-L_{domain}\left(G_{d}\left(R_{\lambda }\left(G_{f}\left(X\right)\right)\right),d\right)$$

where $L_{class}$ and $L_{domain}$ represent the cross-entropy loss functions for sleep stage classification and subject identification, respectively. By optimizing this minimax objective, $G_{f}$ is forced to filter out subject-specific noise, resulting in “F-Common” features that are universally applicable to unseen subjects. This adversarial process ensures that the model focuses on universal physiological biomarkers rather than individual EEG baseline variations.

## 4. Results

This section presents a comprehensive evaluation of the MFST-GCN across multiple dimensions. We begin by describing the datasets and evaluation metrics, followed by implementation details and baseline comparisons. We then systematically analyze model behavior through error pattern visualization, confusion matrices, ablation studies, and module-specific investigations. Finally, we examine computational complexity and parameter sensitivity.

### 4.1. Dataset

We evaluated the MFST-GCN on two subsets of the publicly available ISRUC-Sleep dataset [22], collected between 2009 and 2014 at the Sleep Medicine Center of Coimbra University Hospital following AASM manual recommendations. Polysomnography recordings include multiple physiological signals (EEG, EOG, EMG, ECG) with electrode placement according to the international 10–20 system, covering approximately 8 h overnight sleep sessions.

ISRUC-S1 comprises 100 healthy subjects (mean age: 40.2 ± 16.8 years, 55 females), while ISRUC-S3 contains 10 subjects with suspected sleep disorders (mean age: 53.1 ± 12.4 years, 4 females). Each recording was segmented into 30-second epochs and annotated by two certified polysomnographic technicians into five stages: Wake, N1, N2, N3, and REM. Table 2 presents the stage distribution, revealing natural class imbalance characteristic of sleep architecture—N2 dominates both datasets (31.6% in ISRUC-S1, 30.5% in ISRUC-S3), while N1 represents the minority class (12.7% and 14.2%, respectively).

Data Splitting Strategy: To rigorously evaluate generalization to unseen subjects and avoid data leakage, we employed subject-wise splitting. For ISRUC-S1, subjects were randomly partitioned into 70% training (70 subjects), 15% validation (15 subjects), and 15% testing (15 subjects), ensuring that no subject appears in multiple sets. For ISRUC-S3, given the small sample size, we used leave-one-subject-out cross-validation (LOSO-CV): the model was trained on nine subjects and tested on the remaining subject, repeated 10 times. This protocol simulates real-world clinical deployment where models encounter patients not seen during training.

### 4.2. Evaluation Metrics

We employed multiple complementary metrics to comprehensively assess model performance. Accuracy serves as the primary metric for measuring the proportion of correctly classified instances in sleep stage classification, as defined in Equation (21). Since precision and recall often present a trade-off where it is possible to maximize one at the expense of the other, we employed the F1-score to balance these two metrics, as shown in Equation (22). Additionally, considering the uneven distribution of sleep cycles throughout the night, we utilized Cohen’s kappa coefficient, which is particularly effective for dealing with imbalanced datasets or uneven class distributions, as detailed in Equation (23). The expected agreement by chance denoted as $P_{e}$ in the kappa score is calculated according to Equation (24). In these equations, TP represents the number of true positives where the positive class is correctly predicted as positive, TN represents the number of true negatives where the negative class is correctly predicted as negative, FP represents the number of false positives where the negative class is incorrectly predicted as positive, and FN represents the number of false negatives where the positive class is incorrectly predicted as negative. Precision is defined as $P=\frac{TP}{TP+FP}$ and recall is defined as $R=\frac{TP}{TP+FN}$.

(21) $$Accuracy=\frac{TP+TN}{TP+TN+FP+FN}$$

(22) $$F1-score=2\times \frac{P\times R}{P+R}$$

(23) $$\kappa =\frac{Accuracy-P_{e}}{1-P_{e}}$$

(24) $$P_{e}=\frac{\left(TP+FP)\times (TP+FN)+(FN+TN)\times (FP+TN\right)}{{\left(TP+TN+FP+FN\right)}^{2}}$$

### 4.3. Experimental Setup

The MFST-GCN model was implemented using the TensorFlow 2.0 and Keras frameworks. Experiments were conducted on a computational cluster with four NVIDIA Tesla V100 GPUs (32 GB VRAM each). In this study, we configured the training process with 100 epochs using a batch size of 256. The initial learning rate was set to 0.0003, which is reduced by half every 10 epochs to facilitate convergence. The Adam optimizer was employed for parameter updates, and a dropout rate of 0.5 was applied after major modules to prevent overfitting. All weights in the model were initialized using the uniform method to ensure reproducibility and stable training convergence across different runs.

The MFST-GCN was implemented in TensorFlow 2.x with Keras API. Experiments were conducted on a computational cluster with four NVIDIA Tesla V100 GPUs (32 GB VRAM each). Training used an Adam optimizer with an initial learning rate of 0.0003, decayed by a factor of 0.5 every 10 epochs. The batch size was 256 for ISRUC-S1 and 128 for ISRUC-S3 (adjusted for smaller dataset). The dropout rate of 0.5 was applied after major modules. Weights were initialized using Glorot uniform initialization. Training converged after 100 epochs (approximately 4.2 h for ISRUC-S1 and 1.8 h for ISRUC-S3). The inference time per epoch was 8.2 ms on the GPU, enabling real-time staging at 30 s resolution.

### 4.4. Compared Methods

To validate the effectiveness of the proposed model, we conducted comparative analyses against various state-of-the-art models on the ISRUC_S1 and ISRUC_S3 datasets. The MSTGCN model addresses the challenge of fully leveraging spatial topological information between brain regions by introducing a multi-scale temporal graph convolution network combined with domain generalization for sleep stage classification [23]. The 3D-CNN approach proposes a novel three-dimensional convolutional neural network for sleep stage classification based on multi-channel signals, where the model learns intrinsic connections between different biological signals and frequency bands over time series and spectral dimensions using 3D convolution layers, while employing 2D convolution layers to understand frequency relationships [24].

The JK-STGCN model incorporates two adaptive adjacency matrix learning methods to discern the inherent connections between different biosignal channels for the same and adjacent epochs, utilizing a jumping knowledge spatial–temporal graph convolution network module to learn spatial features with standard convolutions extracting temporal characteristics [18]. The CNN–Transformer–LSTM system combines convolutional neural networks, Transformer, and long short-term memory models to automate sleep stage classification using single-channel EEG signals [25]. The VSTIN architecture proposes a Vision Transformer-based framework to process multi-channel polysomnography signals, capturing spatial information with a pre-trained Vision Transformer and integrating temporal features through self-attention mechanisms [26].

The MFEF model was developed to capture the relational representations between multi-modal physiological signals by combining spectral–temporal and cross-attention representations for sleep stage classification [27]. This network employs a systematic approach to extract spatio-temporal–frequency features and uses cross-attention to merge features from different views effectively. The MVF-SleepNet with SIDA approach addresses significant individual differences among subjects by proposing a structural incentive domain adversarial method, integrating sleep stage classification methods with domain generalization to achieve cross-subject sleep stage classification [28]. Finally, MixSleepNet constructs a novel multi-modal sleep staging model combining 3D convolutional operations with graph convolutions, where the 3D convolution branch explores relationships among multi-channel signals and multiple frequency bands in time series while the graph convolution branch investigates connections between each channel and frequency band [29].

### 4.5. Overall Performance Comparison

Our model underwent comprehensive evaluation for sleep stage classification on the ISRUC_S1 and ISRUC_S3 datasets, and the results are compared with other state-of-the-art models as shown in Table 3 and Table 4. Our model showcased comprehensive superiority over baseline models across multiple evaluation metrics. On the ISRUC_S1 dataset, the MFST-GCN achieved an accuracy of 0.842, F1-score of 0.823, and kappa coefficient of 0.796, representing improvements over the best baseline methods. On the ISRUC_S3 dataset, our model achieved an accuracy of 0.848, F1-score of 0.835, and kappa of 0.804, leading in F1-score and kappa metrics while matching the performance of VSTIN in accuracy. These results validate our model’s efficacy across different sleep staging tasks and demonstrate consistent performance improvements across both datasets.

Notably, shallow depth models like CNN–Transformer–LSTM underperformed on the ISRUC_S1 dataset but showed improved results on the ISRUC_S3 dataset, underscoring the importance of comprehensive training and prediction data for overall model generalization. In relatively shallow models where inputs are primarily in grid format, the dimensions of time and space become crucial due to the dynamic nature of sleep staging. Our proposed deep neural network model demonstrates superior performance on the ISRUC_S1 dataset compared to shallow models, specifically outperforming the MSTGCN, 3D-CNN, and JK-STGCN. Additionally, many deep network models often overlook the interconnections between brain regions. Given the non-Euclidean space of brain regions, our model leveraging brain functional connectivity networks accurately captures spatial relationships within the brain. Consequently, our approach surpasses both spatio-temporal networks and brain functional connectivity networks, achieving state-of-the-art performance across multiple metrics.

Examining the per-stage performance, our model achieved leading F1-scores across Wake, N1, N2, and REM stages on the ISRUC_S1 dataset, and similarly excelled in the N1, N2, and REM categories on the ISRUC_S3 dataset. Specifically, on ISRUC_S1, the MFST-GCN achieved F1-scores of 0.916 for Wake, 0.600 for N1, 0.834 for N2, and 0.873 for REM, all representing the best performance among compared methods. However, the lead in the N1 category was less pronounced compared to other stages, which can be attributed to N1 being a transitional stage between Wake and N2, making it inherently more challenging to classify and more influenced by the staging of adjacent phases. Notably, our model’s performance was slightly lower in the N3 stage, with an F1-score of 0.890 on ISRUC_S1 and 0.906 on ISRUC_S3, where the JK-STGCN achieved 0.901 on ISRUC_S1 and the 3D-CNN achieved 0.909 on ISRUC_S3. This relative underperformance is potentially due to the imbalance in data distribution for the N3 stage, which comprises only 19.8% of total epochs in ISRUC_S1, leading to a bias towards stages with higher proportions of epochs during training.

Our model demonstrates strong generalization performance across ISRUC_S1 and ISRUC_S3 datasets, achieving consistent improvements in accuracy, F1-score, and kappa coefficients. The superior results on both datasets indicate robustness to varying data distributions and sleep stage complexities. Notably, the model excels in most stages except N3, where class imbalance affects performance, highlighting its effective learning from diverse brain connectivity patterns and spatio-temporal features.

### 4.6. Error Pattern Analysis

**Figure 5:**
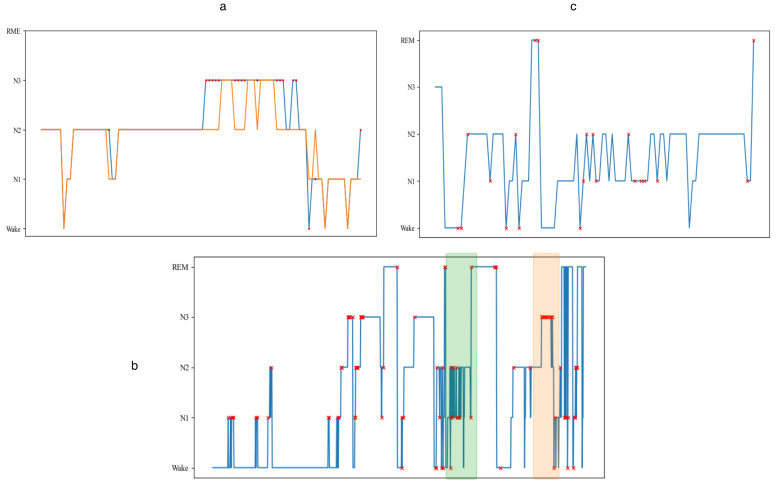
Visualization of sleep stage classification errors across a full night recording. Blue line: ground truth labels. Orange line: predicted labels. Red crosses: misclassification points. (**a**) Full hypnogram. (**b**) Orange-highlighted region: late-cycle N3 instability. (**c**) Green-highlighted region: rapid stage transitions.

As illustrated in Figure 5, we visualized the classification results across a complete sleep recording to understand the error patterns of our model. In this figure, the blue line represents the actual sleep stages of participants while the red cross marks denote misclassifications by our model. The visualization reveals that most misclassifications occur at transition points between different sleep stages, although the overall stability of predictions remains commendable throughout sustained periods of single sleep stages. The expansion of the green section in Figure 5c reveals continuous shifts between sleep stages, demonstrating our model’s capacity to support sleep stability through these transitions by maintaining consistent predictions despite the dynamic nature of sleep stage evolution.

The analysis of the orange section in Figure 5a suggests a decline in performance stability for N3 stages during the latter portion of the sleep cycle. This pattern is possibly due to the model’s incomplete capture of the transition dynamics between N3 and N2 stages as sleep progresses towards awakening. This observation is further corroborated by the rapid increase in REM and Wake stages following this period, indicating the participant’s approaching wakefulness. The concentration of errors at stage boundaries highlights that while our model excels at recognizing stable sleep states, the inherent ambiguity at transition points where physiological signals gradually evolve remains a challenge. This is consistent with the known difficulty that even human experts experience when scoring sleep stages at boundaries, where inter-rater agreement is typically lower than during sustained sleep states.

**Figure 6:**
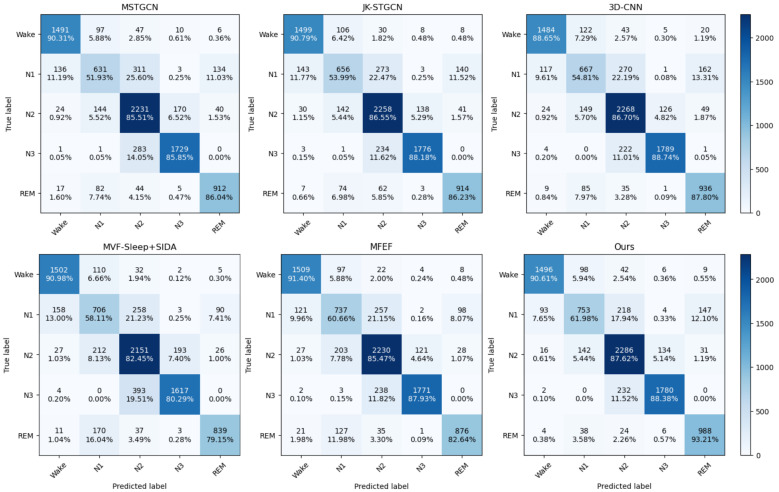
Confusion matrices for sleep stage classification on ISRUC-S3. Each row shows a different model: MFST-GCN (ours), MSTGCN, 3D-CNN, JK-STGCN, MVF-Sleep+SIDA, MFEF. Color intensity indicates proportion of predictions (darker = higher). Diagonal elements represent correct classifications.

Additionally, to further analyze the performance of our proposed model, we visualized the classification accuracy through confusion matrices comparing our model with other state-of-the-art models on the ISRUC_S3 dataset, as shown in Figure 6. The confusion matrix reveals that our model achieved satisfactory results in recognizing the N1, N2, and REM sleep stages, with particularly notable improvements in these indicators compared to baseline methods. This enhancement is attributed to our focus on addressing brain latency effects through the DDFCM module, which allows the model to capture more stable sleep signals by accounting for delayed neural propagation across brain regions, thereby boosting its performance on these challenging stages [30].

However, there was a slight decrease in performance for the easily distinguishable categories of N3 and Wake compared to some specialized baseline methods. This suggests that by enhancing the model’s focus on brain directional information flow and functional connectivity, features from other sleep stages may have been inadvertently introduced, slightly impacting the classification performance of these distinct categories. In the case of the Wake stage, the relatively limited data volume for this class in the ISRUC_S3 dataset, coupled with the model’s effective fitting on REM classification due to their similar EEG patterns, led to some confusion where critical distinguishing features of wakefulness may have been overlooked. Consequently, the classification performance for the Wake stage was marginally compromised, achieving an F1-score of 0.910 compared to CNN–Transformer–LSTM’s 0.942 on ISRUC_S3.

The confusion matrix analysis reveals interesting patterns in misclassifications. The most common confusion occurs between N1 and N2 stages, which is neurophysiologically expected given that N1 represents the transition between wakefulness and established N2 sleep [31]. Similarly, some confusion between Wake and REM is observed, which is understandable given their similar EEG characteristics, including low-amplitude mixed-frequency activity. The N3 stage shows significantly different performance compared to the 3D-CNN multi-modal model. This discrepancy is attributed to the 3D-CNN methodology, which demonstrates that explicitly integrating multi-channel signals with spectral and temporal features yields superior results compared to models that primarily utilize spatial and temporal features, particularly for the N2 and N3 stages, where spectral characteristics like sleep spindles and delta waves are defining features.

In their ablation experiments, the 3D-CNN study showed that the model incorporating only multi-channel features performs comparably to the full spectral–spatial–temporal model specifically in the N2 and N3 stages, further validating the critical importance of explicit spectral feature extraction for these sleep stages. However, this impact does not significantly alter our overall results across all five sleep stages. While enhancing the classification effectiveness of most sleep stages through our functional connectivity and multi-scale attention approach, we acknowledge that the emphasis on spatial–temporal graph features may have intensified certain adverse effects, making the N3 category slightly more susceptible to misclassification in specific scenarios. Moreover, as indicated by the error point distribution in Figure 5, most misclassifications by our model occur at transition points between stages rather than during sustained sleep states, further validating our model’s stability during stable sleep periods and its ability to mitigate the effects of brain information propagation delay through the adaptive lag modeling in the MFST-GCN.

### 4.7. Ablation Study

Table 5 presents systematic ablation experiments on ISRUC-S3 to isolate each module’s contribution. Configuration (a) uses the MSTGCN as baseline (excluding DDFCM, MMFEN, ASTGCN). Configuration (b) omits DDFCM (retains MMFEN, ASTGCN). Configuration (c) removes the MMFEN (retains DDFCM, ASTGCN). Configuration (d) excludes the ASTGCN (retains DDFCM, MMFEN). Configuration (e) employs the full MFST-GCN. Introducing DDFCM alone (Config c vs. a) shows measurable improvements (+0.8% accuracy, +0.6% F1, +1.1% kappa), indicating that functional connectivity modeling enhances performance even without multi-scale processing. DDFCM captures inter-regional connectivity patterns characteristic of different sleep stages. Adding the MMFEN and ASTGCN (Config b, d) further amplifies effectiveness, with Config d (w/o ASTGCN) achieving 0.838 accuracy. The full model (Config e) reaches 0.848 accuracy, demonstrating synergistic effects exceeding individual contributions. The ASTGCN contributes most substantially (+1.0% accuracy over Config d), likely because its position closest to the classifier enables learning task-specific discriminative features. Its multi-kernel design simultaneously captures spatial relationships and temporal dependencies at multiple scales, providing rich representations for classification.

**Figure 7:**
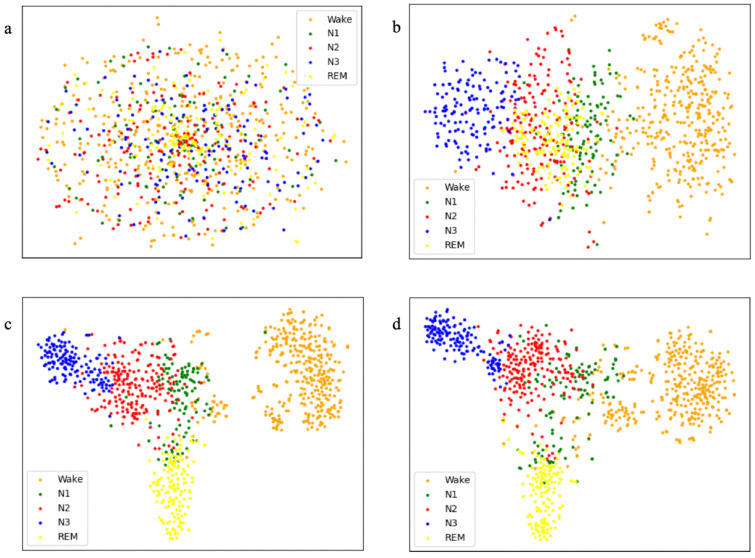
t-SNE visualization of learned feature representations for different model configurations. Each point represents a 30 s epoch, colored by sleep stage (W = Wake, N1–N3 = NREM stages, R = REM). (**a**) Original input features. (**b**) After DDFCM. (**c**) After DDFCM+MMFEN. (**d**) Full model (DDFCM + MMFEN + ASTGCN).

The feature clustering visualization in Figure 7 provides qualitative insights. Figure 7a shows original input features with substantial class overlap and no clear structure, confirming the necessity of sophisticated feature extraction. After DDFCM (Figure 7b), features exhibit initial stratification with partial cluster formation, though boundaries remain blurred. Adding the MMFEN (Figure 7c) creates distinguishable clusters for easily classifiable stages (REM, Wake), but transitional stages remain unclear.

The full model (Figure 7d) achieves distinct, well-separated clusters for Wake, N2, N3, and REM, each occupying compact regions with minimal overlap. N1 appears less separated, showing proximity to the N2 cluster—not a failure but reflecting neurophysiological reality: N1 is transitional, sharing characteristics with both Wake and N2. Similarities between Wake and REM (low-amplitude mixed-frequency EEG) contribute to their relative proximity. These observations corroborate confusion matrix findings: N1 classification couples strongly with N2, followed by Wake.

### 4.8. DDFCM Visualization

**Figure 8:**
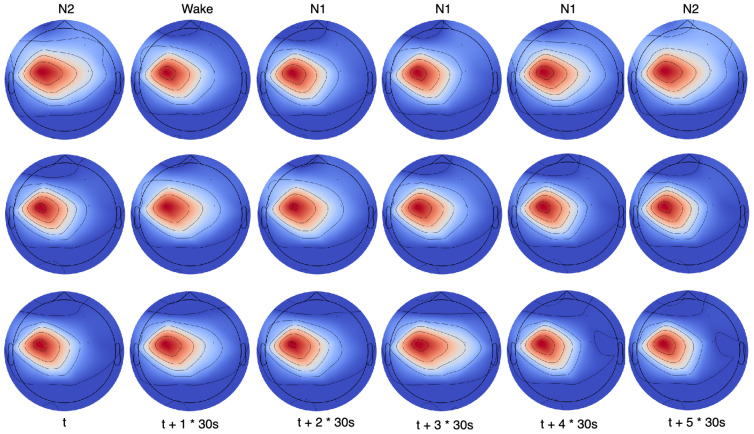
Brain topography map: the top row represents sleep stage categories; the first row shows the brain topography map of the raw data; the second row shows the brain topography map using PCA dimensionality reduction method; the third row shows the brain topography map using t-SNE dimensionality reduction method; and the last row represents the time windows for sleep stage division.

To further explore the efficacy of the DDFCM network in capturing brain functional connectivity patterns and their evolution across sleep stages, we visualized the fused brain topographic maps as shown in Figure 8. This comprehensive visualization displays brain activity patterns across consecutive time windows spanning multiple sleep stages. The figure is organized with the sleep stage type indicated in the top row, and six consecutive 30 s epochs shown in columns below. The visualization employs three different representation methods: the first row shows the brain topography maps derived from raw data without processing, the middle row applies Principal Component Analysis (PCA) for feature dimensionality reduction to capture the primary modes of spatial variation, and the bottom row utilizes t-distributed Stochastic Neighbor Embedding (t-SNE) for nonlinear dimensionality reduction to reveal complex relationships in the high-dimensional brain activity patterns.

From the original brain topographic maps in the raw data row, we can observe that the sleep stage at time point t is labeled as N2, yet the brain activity pattern shows relatively high activation levels that might be suggestive of a transitioning state or approaching awakening. The time point t + 1 clearly shows a wakeful state with widespread activation across multiple brain regions. Subsequently, time points t + 2, t + 3, and t + 4 are all labeled as N1 stages representing the sleep onset period. Notably, at time point t + 4, the raw brain activity appears particularly active and heterogeneous across different regions, indicating a potentially unstable or confused state where the sleep stage characteristics are not clearly defined. This high variability in the raw signal could lead to classification uncertainty and potential errors.

However, after processing and modifications using our proposed DDFCM model as shown in the PCA and t-SNE reduced representations, the brain activity state at time point t+4 becomes substantially more stable and coherent compared to its original state in the raw data. The spatial distribution of activity becomes more consistent with expected N1 characteristics, with the DDFCM module effectively filtering transient noise and artifacts while preserving the underlying sleep-stage-specific patterns. This stabilization occurs because DDFCM constructs functional connectivity graphs that leverage information from multiple brain regions and temporal contexts, allowing the model to distinguish genuine sleep stage characteristics from transient fluctuations. The visualization clearly demonstrates the effectiveness of our functional connectivity-based approach in extracting robust and stable representations that better reflect the true underlying sleep state, even when the raw signals show high variability or ambiguous patterns.

**Figure 9:**
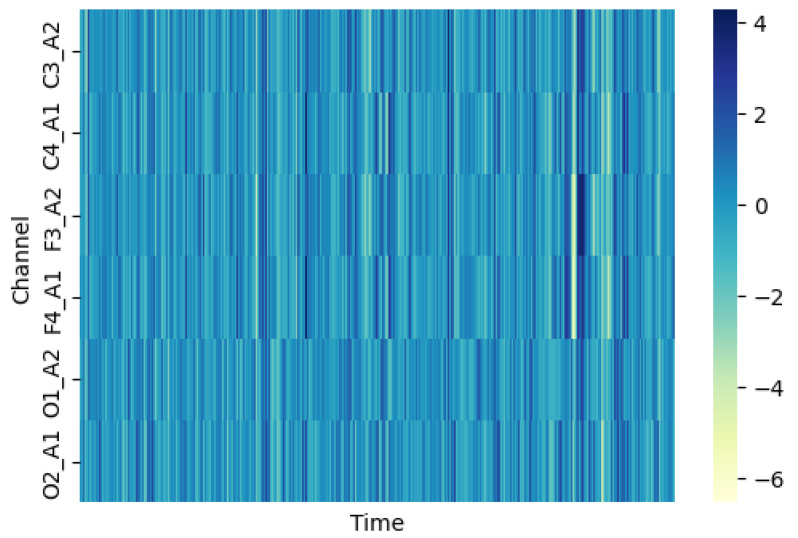
Sleep stage heatmap showing multi-channel EEG amplitude variations within a 30 s epoch. Rows represent channels (F3_A2, C3_A2, O1_A2, etc.). Columns represent time samples. Color intensity indicates normalized amplitude (blue = high, white = low).

Furthermore, to intuitively understand the temporal variation of brain signals within individual sleep stages and across different recording channels, we created a detailed sleep stage signal heatmap as shown in Figure 9. This heatmap visualizes the amplitude variations of multiple EEG channels over time within a single epoch, providing insight into the dynamic patterns that characterize different sleep stages. In this visualization, signal activity is especially prominent in the C3_A2 and F3_A2 channels, which represent central and frontal brain regions, respectively. These channels show sustained high-amplitude activity patterns that are characteristic of the particular sleep stage being displayed. Xin et al. (2022) found significant changes in central and frontal brain regions during sleep [32].

Interestingly, the temporal latency and phase differences in the LOC_A2 channel can be clearly observed in the heatmap. The LOC_A2 channel records activity from the left outer canthus electrode, which primarily captures eye movement artifacts and ocular signals that are particularly relevant for distinguishing REM sleep and Wake stages from NREM stages. The visible latency in this channel’s response relative to central EEG channels demonstrates the propagation delay of neural activity across different brain systems, which is precisely what our DDFCM module is designed to capture and model. The heatmap’s color intensity variations reveal the temporal dynamics of signal propagation and the coordinated activity patterns across multiple recording sites. Brighter colors indicate higher amplitude signals, and the spatial–temporal patterns visible in this visualization reflect the complex interplay between different brain regions during sleep. This type of multi-channel temporal pattern is effectively captured by our model’s functional connectivity approach, which constructs graphs representing the relationships between channels while accounting for the temporal lags that are evident in visualizations like this heatmap.

### 4.9. MMFEN Analysis

**Figure 10:**
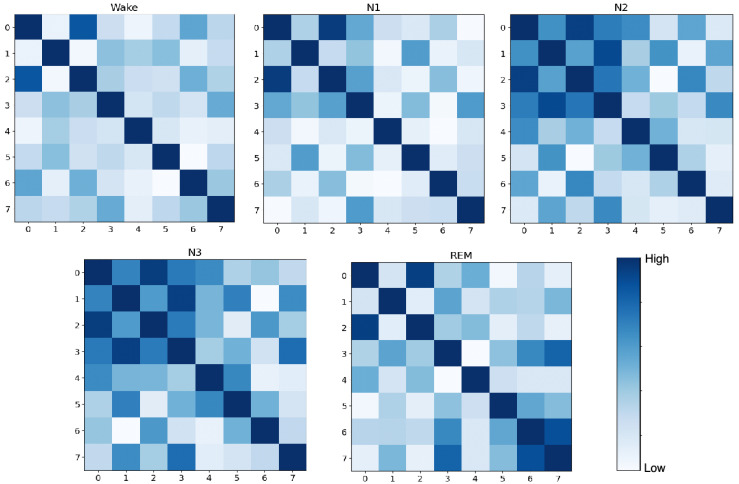
Pearson correlation matrices between EEG channels computed from raw data across five sleep stages. Each subplot shows correlations for one stage (Wake, N1, N2, N3, REM). Color intensity indicates correlation strength (blue = high positive, white = low/negative).

**Figure 11:**
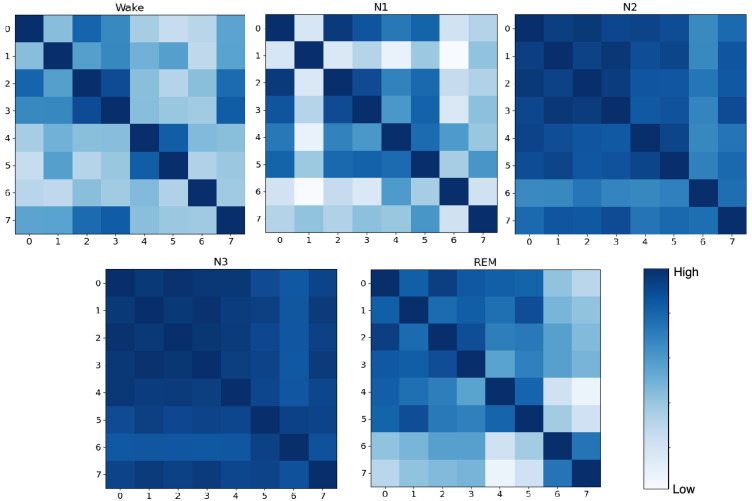
Pearson correlation matrices between EEG channels computed from MMFEN-processed features across five sleep stages. Compared to Figure 10, processed features show enhanced stage-specific connectivity patterns with clearer block structures in N2 and N3.

To delve deeper into the learning effectiveness of the MMFEN component and understand how it differentially weights channels and their relationships across different sleep stages, we visualized and compared the channel-wise feature correlations before and after MMFEN processing. We computed brain channel Pearson correlation matrices that reveal the functional connectivity patterns captured by the model. The original channel correlation matrix derived directly from raw data is presented in Figure 10, while the correlation matrix computed from features after MMFEN processing is shown in Figure 11. Each cell in these matrices represents the Pearson correlation coefficient between a pair of channels across all epochs of a particular sleep stage category.

A larger Pearson coefficient in these matrices suggests more consistent and synchronized signal variations between two channels across time, implying tighter functional connectivity or more coherent information transfer between the corresponding brain regions. As clearly shown in Figure 10, the Wake stage exhibits the most extensive brain functional connections among all sleep stages, with high correlation values distributed widely across the correlation matrix. This pattern reflects the active and coordinated neural activity across multiple brain regions that characterizes the wakeful state, where sensory processing, attention, and voluntary control systems are simultaneously engaged.

Among the three NREM sleep stages, N1, being the initial stage of sleep onset, often shows relatively active brain functionality compared to deeper sleep stages. As a transitional stage between wakefulness and consolidated sleep, N1 is characterized by complex and somewhat unstable neural dynamics, making it the most intricate sleep stage with numerous functional connections visible in the correlation matrix. The heterogeneous connectivity pattern in N1 reflects the brain’s gradual disengagement from wake-like processing while not yet fully entering the synchronized patterns of deeper NREM sleep. In contrast, the N3 stage demonstrates the fewest and weakest inter-channel correlations during sleep, which is consistent with the high-amplitude, low-frequency delta activity that dominates this deep sleep stage. During N3, the brain exhibits more localized and less coordinated activity patterns as reflected in the sparser correlation structure.

REM sleep presents a distinct and intermediate correlation pattern that reflects its unique neurophysiological characteristics. As a stage that typically occurs in the latter half of the sleep cycle following N2 and N3 stages, REM sleep combines features of both deep sleep and wakefulness, with cortical activation patterns resembling wakefulness while muscle tone remains suppressed. Consequently, the functional connectivity structure during REM occupies an intermediate position between the synchronized patterns of N2 or N3 and the diverse connectivity of Wake stages, as evidenced by the moderate correlation strengths and distribution patterns in the matrix.

Figure 11 presents the correlation matrices after MMFEN processing, which further clarifies and enhances these stage-specific connectivity trends. The MMFEN module effectively learns to emphasize functionally relevant connections while suppressing spurious correlations that may arise from noise or artifacts in the raw data. Notably, the processed features show tighter and more distinct connectivity patterns between channels during the N2 and N3 stages compared to the raw data correlations. This enhancement indicates that the MMFEN successfully extracts the essential functional connectivity signatures that distinguish these deep sleep stages. The multi-scale attention mechanism achieves this by simultaneously analyzing channel relationships at different temporal resolutions, allowing it to capture both fast transient events and slower evolving sleep stage characteristics. The clearer block structure visible in the MMFEN-processed correlation matrices for N2 and N3 suggests that the attention mechanism has learned to group channels into functionally coherent networks that reflect the underlying neurophysiology of consolidated NREM sleep.

### 4.10. Channel Importance Analysis

**Figure 12:**
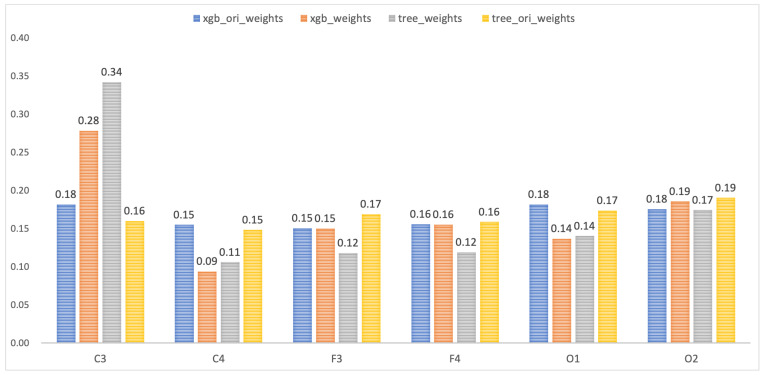
Channel importance weights under different classification models. Left: xgb_ori_weights (XGBoost on raw data). Right: xgb_weights (XGBoost on ASTGCN features). Bars show relative importance of each channel (F3_A2, C3_A2, O1_A2, etc.) across five sleep stages.

Figure 12 presents channel importance analysis using feature attribution methods to understand how different channels contribute to model decisions throughout sleep cycles. After extracting deep features from the ASTGCN module, we applied t-SNE dimensionality reduction to project high-dimensional representations into lower-dimensional space suitable for downstream analysis. We then trained two tree-based classifiers (XGBoost and DecisionTreeClassifier) on these reduced features to perform sleep stage classification. By examining feature importance scores, we can understand which channels carry most discriminative information for sleep staging after being processed by our model.

In Figure 12, xgb_ori_weights represents the feature weight map obtained from applying the XGBoost classifier directly to dimensionality-reduced original raw data without ASTGCN processing, serving as a baseline for comparison. In contrast, xgb_weights shows the feature weight map obtained from features extracted by the ASTGCN module before classification. Comparison between these distributions reveals the effect of our spatial–temporal graph convolution processing on channel importance patterns. Each channel exhibits distinct varying feature weights across different sleep stages, which is expected given that different brain patterns and activity distributions characterize each sleep stage. Stage-specific importance patterns reflect varying roles that different brain regions play during wakefulness, light sleep, deep sleep, and REM sleep.

Most notably, feature weight on the C3 channel (recording activity from central brain region) increases by approximately 10 percentage points after processing through the ASTGCN module compared to the raw data baseline. This substantial increase highlights the richness and relevance of physiological information captured in the C3 channel, which is anatomically situated at the scalp’s mid-central layer overlying the primary motor and somatosensory cortex. The C3 location is particularly well-suited for capturing sleep-related rhythms, including sleep spindles, K-complexes, and the waxing and waning of delta activity that characterize different NREM stages. The enhanced importance of C3 after ASTGCN processing suggests that our multi-kernel spatial–temporal graph convolution effectively amplifies and refines sleep-relevant information from this critical channel while integrating it with complementary information from other channels.

Brain topographic visualizations presented earlier in Figure 8 also underscore and corroborate the importance of the C3 channel in sleep staging. In those topographic maps, the C3 region consistently shows distinctive and stable activity patterns that evolve systematically across different sleep stages. The convergence of evidence from both feature importance analysis and spatial activity visualizations strongly supports conclusion that the C3 channel provides particularly valuable information for automatic sleep stage classification, and that our ASTGCN module successfully learns to leverage this information through its graph-based spatial–temporal feature extraction mechanism. The multi-kernel design allows the model to capture C3’s relationships with other channels at multiple time scales, from fast sleep spindles to slow delta rhythms, maximizing the utility of this information-rich recording site.

### 4.11. Computational Complexity Analysis

Table 6 presents time complexity analysis comparing the MFST-GCN with baseline methods. To ensure fair comparison, all models were evaluated on the ISRUC_S3 dataset under identical hardware and software configurations. In this analysis, E represents the number of training epochs, B the number of batches per epoch, M the batch size, L the number of 3D convolutional layers, L’ the number of 2D convolutional layers, T the number of time steps, V the number of vertices (channels), F the number of features, K the convolution kernel size, N the total number of data samples, BL the number of graph convolutions, and k the order of Chebyshev polynomial.

Since our method extends the MSTGCN architecture, it involves a greater number of network parameters, leading to higher theoretical time complexity relative to the MSTGCN baseline. However, this increase is justified by the superior model performance achieved. Compared to MFEF, the time complexity of our model is nearly identical (both O(BL × ${\mathrm{TVF}}^{2}$×(1+k+V) order)), yet our model demonstrates better performance metrics. MFEF incorporates both time–frequency and graph learning networks, as well as VGG-16 and GRU networks, contributing to the overall computational cost.

In contrast, when compared with 3D-CNN models, our approach exhibits advantages in both performance and time complexity. The increased time complexity in 3D-CNN models stems from the inclusion of higher-dimensional 3D convolutional networks, which significantly increase the overall computational cost due to cubic convolution kernel operations ($K^{3}$ term). Our graph-based approach achieves superior performance while maintaining computational efficiency through sparse connectivity patterns inherent in brain functional networks, avoiding dense 3D convolutions.

Empirical training times on the ISRUC_S3 dataset corroborate theoretical analysis. The MFST-GCN required 1.8 h for 100 epochs, comparable to MFEF (1.9 h) and significantly faster than the 3D-CNN (2.8 h), while achieving the best performance metrics. The inference time per 30 s epoch was 8.2 ms on the Tesla V100 GPU, enabling real-time sleep staging applications. In summary, the increase in time complexity of our model remains within an acceptable range given improvements in model performance, maintaining practical feasibility for clinical deployment.

### 4.12. Parameter Sensitivity Analysis

**Figure 13:**
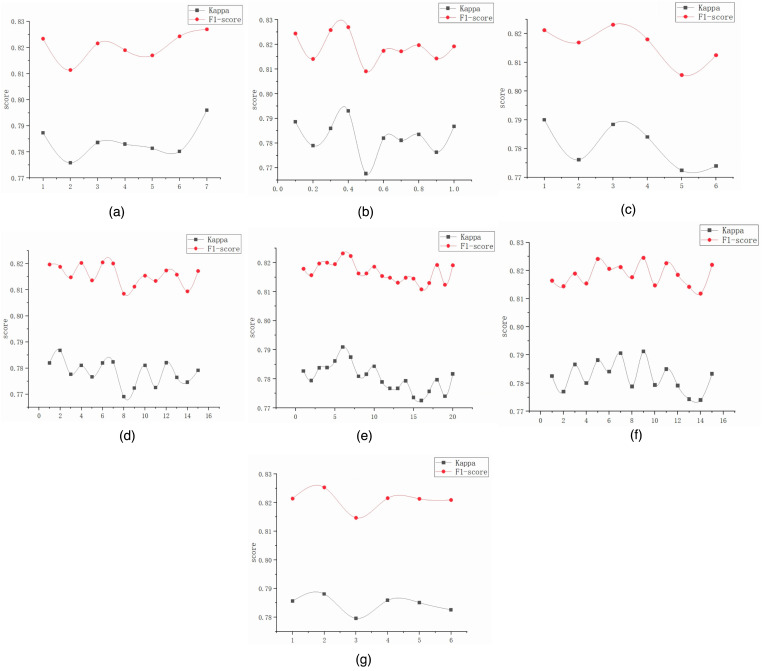
Parameter sensitivity analysis showing F1-score and kappa across different configurations. (**a**) Domain generalization loss weight alpha. (**b**) Multi-scale attention kernel configurations. (**c**–**g**) Spatial–temporal graph convolution kernel numbers (1 to 5 kernels).

Figure 13 investigates the impact of key hyperparameters on model performance, with kappa coefficient and F1-score metrics visualized across parameter variations. To ensure validity, all experiments were conducted on an ISRUC_S3 dataset with identical protocols, varying only the parameter under investigation while keeping others constant.

First, we explored domain generalization loss weight parameter alpha, as shown in Figure 13a. Experiments with alpha values ranging from 0.1 to 1.0 revealed that optimal performance occurred at an alpha equal to 0.4, achieving an F1-score of 0.835 and kappa of 0.804. The performance variation across the tested range was approximately 1 percentage point, indicating reasonable robustness around the optimal value. Values below 0.3 resulted in insufficient domain adaptation, while values above 0.6 caused excessive focus on the adversarial objective at the expense of classification accuracy. This validates yjr effectiveness of domain adversarial training while demonstrating that moderate weighting balances discriminative power and domain invariance.

Second, we examined multi-scale attention kernel configurations in the MMFEN module, dividing six kernel sizes (50, 100, 200, 400, 700, 1000 samples) into different combinations for systematic validation. Figure 13b shows that the configuration X equals 7 (combining all six kernel sizes) achieved a superior performance, with an F1-score of 0.835 and kappa of 0.804. Both metrics exhibited an initial decline followed by a gradual increase, with particularly significant improvements at configurations of X equals 6 and 7. Configuration X equals 1 (single kernel) achieved an F1 of 0.812 and kappa of 0.763, while the progressive addition of kernels monotonically improved performance. This confirms that integrating multiple convolutional kernels at different receptive field sizes effectively captures sleep-relevant patterns at multiple temporal scales, from fast spindles to slow waves.

Third, we investigated the impact of different numbers of spatial–temporal graph convolution kernels in the ASTGCN module, with the results presented in Figure 13c–g. Single-kernel networks (Figure 13c) achieved the best results at configuration X equals 3, with an F1 of 0.818 and kappa of 0.771, demonstrating that even single-scale graph convolution provides substantial improvement over the baseline. Dual-kernel fusion (Figure 13d) showed limited incremental improvement (F1: 0.822, kappa: 0.778). However, three-kernel (Figure 13e) and four-kernel (Figure 13f) configurations demonstrated substantial performance gains, with the F1 reaching 0.828 and 0.832, respectively. Configurations X equals 6 and 7 yielded notably better results, approaching full model performance.

The five-kernel configuration (Figure 13g) showed diminishing returns, with the F1 plateauing around 0.833, suggesting a saturation point beyond which additional kernels provide a marginal benefit while increasing the model complexity. Ultimately, six-kernel fusion achieved the most significant enhancement across both metrics (F1: 0.835, kappa: 0.804), confirming that multiple kernels with different receptive fields effectively capture complementary aspects of brain functional connectivity dynamics while maintaining an optimal balance between representation capacity and model complexity. Beyond six kernels, the computational cost increases without commensurate performance gains, validating our architectural choice.

These parameter sensitivity analyses demonstrate that the MFST-GCN achieves a robust performance across reasonable parameter ranges, with optimal configurations identified through systematic ablation. The multi-scale nature of both MMFEN (temporal scales) and ASTGCN (spatial–temporal scales) modules is validated through monotonic performance improvements as scale diversity increases, up to a saturation point balancing capacity and complexity.

## 5. Discussion

The proposed MFST-GCN framework demonstrates significant advantages in sleep stage classification through multi-scale, dynamic, and adaptive modeling strategies that effectively capture time-lag effects and regional heterogeneity in EEG signals. This section synthesizes our findings, relates them to neurophysiological principles, compares against prior work, acknowledges limitations, and suggests future directions.

### 5.1. Key Findings and Neurophysiological Interpretation

Our experimental results validate three central hypotheses. First, modeling brain functional connectivity at dual timescales (1 s and 5 s windows in DDFCM) significantly improves the recognition of transitional sleep stages, particularly N1 and N2. As demonstrated in ablation studies (Table 5), incorporating DDFCM alone improved the F1-score by 0.6 percentage points over the baseline, with gains concentrated in N1 classification. This aligns with neurophysiological evidence that sleep stage transitions involve both rapid desynchronization events (captured by 1 s windows) and sustained reconfiguration of large-scale connectivity patterns (captured by 5 s windows) [30]. Traditional static graph models fail to capture these temporal dynamics, leading to confusion at stage boundaries, where our model excels.

Second, multi-scale morphological feature extraction (MMFEN) addresses spatial heterogeneity in brain activation patterns. Parameter sensitivity analysis (Figure 13b) showed monotonic performance improvement as the kernel diversity increased, confirming that different brain regions exhibit rhythms at distinct timescales. Central regions (C3_A2, C4_A1) show prominent slow-wave activity (0.5–4 Hz, corresponding to 700–1000 sample kernels at 200Hz), while frontal regions (F3_A2, F4_A1) exhibit faster spindle oscillations (11–16 Hz, corresponding to 50–100 sample kernels). By extracting features at multiple scales simultaneously, the MMFEN captures stage-specific signatures (e.g., sleep spindles in N2, delta waves in N3) more effectively than fixed-scale approaches.

Third, adaptive spatial–temporal graph convolution (ASTGCN) with a multi-kernel design enhances the model capacity to encode global dependencies while maintaining computational efficiency. Channel importance analysis (Figure 12) revealed that the ASTGCN amplifies the discriminative power of critical channels (e.g., C3 importance increased by 10 percentage points), consistent with prior studies showing central regions’ pivotal role in sleep regulation [32]. The multi-kernel architecture captures both fast local synchronization (small receptive fields) and slow inter-regional coordination (large receptive fields), mirroring the hierarchical organization of sleep-regulating brain networks.

### 5.2. Comparison with Prior Work

Our framework advances beyond existing approaches in several key aspects. Compared to static graph models (MSTGCN, JK-STGCN), the MFST-GCN incorporates dynamic connectivity modeling through DDFCM, accounting for time-lag effects in neural signal propagation [31]. While the MSTGCN achieved a kappa of 0.765 on ISRUC-S3, our model reached 0.804 (5.1% relative improvement), primarily driven by better N1 classification (0.650 vs. 0.581 F1-score). This validates our hypothesis that explicit temporal lag modeling is crucial for transitional stage recognition.

Compared to multi-modal spectral–temporal models (3D-CNN, MFEF), the MFST-GCN achieves competitive or superior performance while maintaining lower computational complexity. The 3D-CNN incorporates explicit frequency domain features through 3D convolutions over spectral–spatial–temporal dimensions, yielding strong N3 performance (F1: 0.909 on ISRUC-S3). However, their ablation studies showed that spectral features primarily benefit N2 and N3 stages, where frequency characteristics (spindles, K-complexes) are defining markers [24]. Our approach captures similar information through multi-scale temporal convolutions in the MMFEN, which implicitly extract frequency-specific patterns without expensive 3D operations, resulting in 1.8-h training versus the 3D-CNN’s 2.8 h on ISRUC-S3.

MFEF employs cross-modal attention to fuse time–frequency and graph representations, achieving a kappa of 0.785 on ISRUC-S3. While our model’s kappa (0.804) represents modest improvement, the key distinction lies in architectural philosophy: MFEF treats temporal and spatial features as separate modalities requiring late fusion, whereas the MFST-GCN integrates them through a unified graph convolution framework. This enables end-to-end learning of spatial–temporal dependencies without modality-specific encoders, improving parameter efficiency (Table 6).

Domain generalization methods (MVF-SleepNet+SIDA) achieve cross-subject robustness through adversarial training. Our ADG module adopts a similar strategy but integrates it within a graph-based framework, yielding superior performance (0.804 vs. 0.738 kappa). The combination of functional connectivity modeling and domain adversarial learning appears synergistic: connectivity graphs encode subject-invariant brain topology, while adversarial training aligns feature distributions across subjects. This suggests that graph representations provide a better substrate for domain adaptation than the grid-based features used in MVF-SleepNet.

### 5.3. Limitations and Future Directions

Despite encouraging results, several limitations warrant discussion. First, our model shows relative underperformance in the N3 stage (F1: 0.906 on ISRUC-S3) compared to specialized methods like the 3D-CNN (F1: 0.909). This stems from class imbalance (N3 constitutes only 23.6% of ISRUC-S3 epochs) and our emphasis on spatial–temporal graph features potentially diluting explicit spectral characteristics. Future work could integrate frequency-domain priors (e.g., wavelet transforms, power spectral density) as auxiliary inputs to the MMFEN, combining strengths of graph-based spatial modeling and explicit spectral analysis. Alternatively, class-balanced sampling strategies or focal loss could mitigate imbalance effects during training.

Second, interpretability remains limited despite visualization efforts (Figure 7, Figure 8, Figure 10, and Figure 11). While t-SNE projections and correlation matrices provide post hoc insights, they do not fully explain how specific architectural components (e.g., Chebyshev graph filters, attention mechanisms) contribute to individual predictions. Future research could incorporate inherently interpretable modules such as prototype learning [33] or concept bottleneck models [34], enabling clinicians to understand why the model classified an epoch as a specific stage. Attention weight visualizations could be extended to show which channel pairs and temporal windows most influenced each decision.

Third, generalization to diverse clinical populations and recording protocols requires further validation. ISRUC datasets contain primarily healthy subjects (ISRUC-S1) or suspected sleep disorder patients (ISRUC-S3), but lack representation of specific conditions (e.g., severe sleep apnea, REM behavior disorder, pediatric populations). Transfer learning experiments across datasets (e.g., training on ISRUC, testing on Sleep-EDF, MASS) would assess robustness to distribution shift. Additionally, our model uses 6-channel EEG following the ISRUC protocol, whereas clinical practice varies (single-channel wearables to high-density 128-channel systems). Developing channel-agnostic architectures through graph neural network variants (e.g., graph attention networks allowing for variable node sets) could enhance practical applicability.

Fourth, computational efficiency, while improved over the 3D-CNN, remains a barrier for real-time edge deployment on resource-constrained devices (e.g., wearable sleep monitors). Model compression techniques (pruning, quantization, knowledge distillation) could reduce inference latency from 8.2 ms to the sub-millisecond range without significant performance degradation. Alternatively, lightweight graph convolution variants (e.g., simplified graph convolutions, MobileNet-inspired factorizations) could maintain accuracy while enabling on-device inference.

Fifth, clinical deployment challenges extend beyond algorithmic performance. Regulatory approval (FDA, CE marking) requires extensive validation, including inter-rater agreement studies comparing model outputs against certified technicians’ consensus scoring. Uncertainty quantification through Bayesian deep learning or ensemble methods could provide confidence estimates for predictions, enabling clinicians to identify ambiguous epochs requiring a manual review. The user interface design for presenting automated staging results alongside raw PSG signals, with mechanisms for expert correction and model retraining, represents a critical human–computer interaction research direction.

Sixth, the biological plausibility of dual-timescale connectivity modeling (1 s and 5 s windows) deserves further scrutiny. While our empirical results support this choice, optimal timescales may vary across sleep stages, individuals, or pathological conditions. Adaptive windowing strategies that learn stage-specific or subject-specific temporal scales through meta-learning could enhance flexibility. Neurophysiological validation through simultaneous EEG-fMRI or intracranial recordings would provide a ground truth for connectivity dynamics, enabling biophysically informed architecture design.

### 5.4. Clinical Implications and Future Applications

Beyond technical contributions, the MFST-GCN holds promise for advancing sleep medicine practice. Automated staging can reduce the expert workload, enabling large-scale epidemiological studies previously limited by manual scoring costs. Our model’s superior N1 classification (F1: 0.650 vs. 0.629 for the best baseline on ISRUC-S3) is clinically significant, as N1 abnormalities often indicate early-stage neurodegenerative diseases (e.g., Parkinson’s, Alzheimer’s) where the REM sleep behavior disorder precedes motor symptoms by decades [31]. Enhanced sensitivity to subtle N1 changes could enable earlier diagnosis and intervention.

The framework’s modular design facilitates extensions to related tasks. DDFCM’s functional connectivity graphs could inform network-based biomarkers for sleep disorders, revealing disrupted connectivity patterns in insomnia, sleep apnea, or circadian rhythm disorders. The MMFEN’s multi-scale features could be adapted for the automated detection of sleep-related events (apneas, arousals, periodic leg movements), replacing labor-intensive manual annotation. The ASTGCN’s spatial–temporal encoding could transfer to other neurophysiological time series (e.g., seizure detection in epilepsy, cognitive state decoding in brain–computer interfaces).

Longitudinal monitoring applications represent a particularly exciting frontier. Home-based sleep studies using simplified EEG montages could benefit from our graph-based approach, which inherently handles variable channel sets through flexible graph construction. Tracking connectivity dynamics across nights could reveal sleep architecture evolution during treatment (e.g., CPAP therapy for apnea, cognitive behavioral therapy for insomnia), providing objective biomarkers of therapeutic response. Integration with wearable sensors (actigraphy, heart rate variability, respiratory effort) through multi-modal graph fusion could enable comprehensive sleep phenotyping beyond traditional staging.

## 6. Conclusions

In this paper, we introduce a novel deep graph neural network model, the MFST-GCN, for sleep stage classification. Addressing brain lag effects, the MFST-GCN model thoroughly considers the intracerebral information flow and brain region activity patterns. Specifically, we designed the sleep brain functional connectivity network to accommodate the varying time spans of intracerebral information flow. Furthermore, the multi-scale attention mechanism introduced enhances the integration of multi-scale features. Additionally, a multi-scale spatio-temporal graph convolution network was developed to capture features across different temporal and spatial dimensions. Finally, to enhance the model’s robustness, we incorporated a domain generalization approach, integrating it with the multi-scale spatio-temporal graph convolution network within a unified framework. Our experiments on two public datasets, ISRUC_S1 and ISRUC_S3, demonstrate the model’s effectiveness, showcasing superior performance across various metrics.

It is important to note that while the proposed method has shown improvements in sleep stage classification, it is not yet suitable for clinical application. Further collaboration with hospitals will be necessary to translate this model into a clinically applicable system. Moreover, sleep disorders frequently disrupt sleep, significantly impacting sleep quality. To address a broader range of domain-specific variations, our future work will focus on leveraging large language models to develop more generalizable and robust approaches. The goal is to enhance sleep stage classification performance in the context of sleep disorders.

## Figures and Tables

**Table 1:** Module specifications and functional roles in MFST-GCN.

Module	Input	Output	Purpose
DDFCM	Raw EEG signals (6 channels × 6000 samples)	Connectivity embedding (128-dim)	Model propagation delays via dual-scale correlation graphs
MMFEN	Raw EEG signals (6 channels × 6000 samples)	Multi-scale features (384-dim)	Extract frequency-specific patterns across temporal resolutions
ASTGCN	Concatenated features (512-dim)	Spatio-temporal embedding (256-dim)	Integrate spatial dependencies and temporal evolution
ADG	Final embedding (256-dim)	Class probabilities (5-dim)	Enforce subject-invariant representations

**Table 2:** Distribution of sleep stages across ISRUC datasets. Natural class imbalance reflects physiological sleep architecture, with N2 constituting the largest proportion and N1 the smallest.

Dataset	Subjects	Total Epochs	Wake	N1	N2	N3	REM
ISRUC-S1	100	87,187	20,098 (23.1%)	11,062 (12.7%)	27,511 (31.6%)	17,251 (19.8%)	11,265 (12.9%)
ISRUC-S3	10	8549	1651 (19.3%)	1215 (14.2%)	2609 (30.5%)	2014 (23.6%)	1060 (12.4%)

**Table 3:** Results of the ISRUC_S1 dataset.

Method	Accuracy	F1-Score	Kappa	Wake	N1	N2	N3	REM
MSTGCN	0.808	0.787	0.752	0.885	0.539	0.799	0.876	0.838
3D-CNN	0.820	0.797	0.768	0.908	0.534	0.808	0.880	0.855
JK-STGCN	0.831	0.814	0.782	0.900	0.598	0.826	0.901	0.845
CNN–Transformer–LSTM	0.804	0.772	0.750	0.908	0.498	0.801	0.885	0.772
VSTIN	0.829	0.777	0.775	-	-	-	-	-
MFEF	0.821	0.799	0.769	0.905	0.583	0.810	0.889	0.851
MVF-SleepNet + SIDA	0.794	0.777	0.734	0.878	0.539	0.782	0.857	0.831
MixSleepNet	0.829	0.791	0.775	0.903	0.482	0.826	0.878	0.864
ours	0.842	0.823	0.796	0.916	0.600	0.834	0.890	0.873

**Table 4:** Results of the ISRUC_S3 dataset.

Method	Accuracy	F1-Score	Kappa	Wake	N1	N2	N3	REM
MSTGCN	0.818	0.803	0.765	0.898	0.581	0.808	0.880	0.848
3D-CNN	0.832	0.814	0.783	0.896	0.596	0.862	0.909	0.838
JK-STGCN	0.820	0.798	0.767	0.895	0.550	0.811	0.886	0.850
CNN–Transformer–LSTM	0.824	0.815	0.770	0.942	0.629	0.810	0.856	0.837
VSTIN	0.840	0.796	0.781	-	-	-	-	-
MFEF	0.833	0.824	0.785	0.906	0.619	0.826	0.905	0.846
MVF-SleepNet + SIDA	0.797	0.788	0.738	0.896	0.585	0.785	0.844	0.831
MixSleepNet	0.838	0.820	0.790	0.891	0.620	0.833	0.904	0.853
ours	0.848	0.835	0.804	0.910	0.650	0.838	0.906	0.869

**Table 5:** Ablation study results on ISRUC-S3 dataset. Each row represents a configuration with different module combinations. Checkmarks indicate included modules.

Config	DDFCM	MMFEN	ASTGCN	Accuracy	F1-Score	Kappa
(a) Baseline	-	-	-	0.818	0.803	0.765
(b) w/o DDFCM	-	✔	✔	0.832	0.816	0.784
(c) w/o MMFEN	✔	-	✔	0.826	0.809	0.776
(d) w/o ASTGCN	✔	✔	-	0.838	0.821	0.790
(e) Full Model	✔	✔	✔	0.848	0.835	0.804

**Table 6:** Time complexity comparison on ISRUC_S3 dataset. Our model shows comparable complexity to MFEF while outperforming 3D-CNN in both efficiency and accuracy.

Method	Time Complexity	Training Time (h)
MSTGCN	O(BL × ${\mathrm{TVF}}^{2}$ × (1+k+V))	1.5
3D-CNN	O(E × B × (*L* × (${\mathrm{TV}}^{2}$ $F^{2}$ $K^{3}$ + ${\mathrm{TVF}}^{2}$ + N) + $L^{\prime }$ × (${\mathrm{TV}}^{2}$ $F^{2}$ $K^{2}$ + ${\mathrm{TVF}}^{2}$ + N)))	2.8
MFEF	O(BL × ${\mathrm{TVF}}^{2}$ × (1+k+V))	1.9
Ours (MFST-GCN)	O(BL × (${\mathrm{TVF}}^{2}$ + ${\mathrm{TVkF}}^{2}$ + ${\mathrm{TV}}^{2}$ F))	1.8

## Data Availability

No datasets were generated or analyzed during the current study. The corresponding code will be published on https://github.com/lihuifu/MFST-GCN (accessed on 26 January 2026).
